# Lenacapavir: First Approval

**DOI:** 10.1007/s40265-022-01786-0

**Published:** 2022-10-22

**Authors:** Julia Paik

**Affiliations:** Springer Nature, Mairangi Bay, Private Bag 65901, Auckland, 0754 New Zealand

## Abstract

Lenacapavir (Sunlenca^®^) is a long-acting capsid inhibitor of human immunodeficiency virus type 1 (HIV-1) being developed by Gilead Sciences Inc. It is available as an oral tablet and injectable solution, with the latter being a slow-release formulation to allow bi-annual subcutaneous administration. In August 2022, lenacapavir received its first approval in the EU for use in combination with other antiretroviral(s) in adults with multi-drug resistant HIV infection, for whom it is otherwise not possible to construct a suppressive anti-viral regimen. This article summarizes the milestones in the development of lenacapavir leading to this first approval for the treatment of HIV-1 infection.

**Supplementary Information:**

The online version contains supplementary material available at 10.1007/s40265-022-01786-0.

**Table:** 

**Digital Features** for this AdisInsight Report can be found at https://doi.org/10.6084/m9.figshare.21183850	

## Lenacapavir (Sunlenca^®^): Key Points

**Table:** 

A long-acting HIV capsid inhibitor allowing bi-annual SC administration is being developed by Gilead Sciences Inc. for the treatment of HIV-1 infection
Received its first approval on 22 August 2022 in the EU
Approved in the EU for use in combination with other antiretroviral(s) in adults with multi-drug resistant HIV infection, for whom it is otherwise not possible to construct a suppressive anti-viral regimen

## Introduction

Infection with human immunodeficiency virus type 1 (HIV-1) can typically be managed with antiretroviral treatments, including combination antiretroviral therapy, which may increase the life expectancy of patients with HIV-1 to levels comparable to that of the general population [1]. However, treatment success relies on proper treatment adherence, the lack of which contributes to the development of viral resistance [2]. Barriers to adherence in the context of HIV-1 treatments include tolerability issues and social factors such as stigma and lack of support, in addition to factors relating to convenience (e.g. forgetting treatment, change in routine) [3]. As such, durable and convenient treatments requiring less frequent administration that can facilitate high adherence are crucial to successfully manage the disease, particularly in those with multi-drug resistant HIV.

Lenacapavir (Sunlenca^®^) is a long-acting HIV capsid inhibitor being developed by Gilead Sciences Inc. for the treatment of HIV-1 infection, formulated as an oral tablet and an injectable solution [4]. Lenacapavir inhibits viral replication at both the early and late stages of the HIV life cycle, and its injectable formulation is designed to slowly release the drug from the injection site to allow bi-annual subcutaneous administration. On 22 August 2022 [5], lenacapavir was approved in the EU for use in combination with other antiretroviral(s) in adults with multi-drug resistant HIV infection, for whom it is otherwise not possible to construct a suppressive anti-viral regimen [4].

**Figure:**
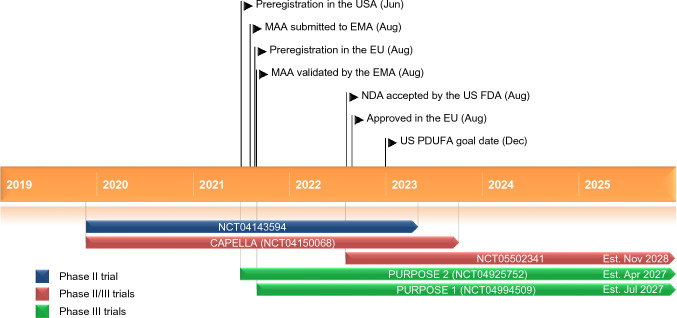
Key milestones in the development of lenacapavir for the treatment of HIV-1 infections. *EMA* European Medicines Agency, *FDA* Food and Drug Administration, *MAA* Marketing Authorization Application, *NDA* New Drug Application, *PDUFA* Prescription Drug User Fee Act

When initiating treatment, oral lenacapavir 600 mg/day is recommended for days 1 and 2, and lenacapavir 300 mg on day 8; on day 15, lenacapavir 927 mg should be administered via subcutaneous (SC) injection. For maintenance treatment, SC lenacapavir 927 mg should be administered once every 6 months from the date of the last injection. No dose adjustments of lenacapavir is required in patients with mild or moderate hepatic impairment, nor in those with mild, moderate, or severe kidney impairment. Lenacapavir use has not been studied in patients with end-stage kidney disease [creatinine clearance (CrCl) < 15 mL/min]; caution is needed if lenacapavir is used in these patients. Its use is contraindicated in patients also receiving strong inducers of CYP3A, P-glycoprotein, and UDP-glucuronosyltransferase 1A1 [4].

### Company Agreements

In March 2021, Gilead Sciences Inc. and Merck Sharp & Dohme entered into an agreement to jointly co-develop and co-commercialize lenacapavir and islatravir (a nucleoside reverse transcriptase translocation inhibitor) for the treatment of patients with HIV [6]. The initial focus of this collaboration is on long-acting oral and long-acting injectable formulations of these combination products, with other formulations potentially added to the collaboration as mutually agreed. Beyond the potential combinations of lenacapavir and islatravir, Gilead Sciences Inc. will also have the option to license certain investigational oral integrase inhibitors from Merck Sharp & Dohme to develop in combination with lenacapavir [6].

**Figure:**
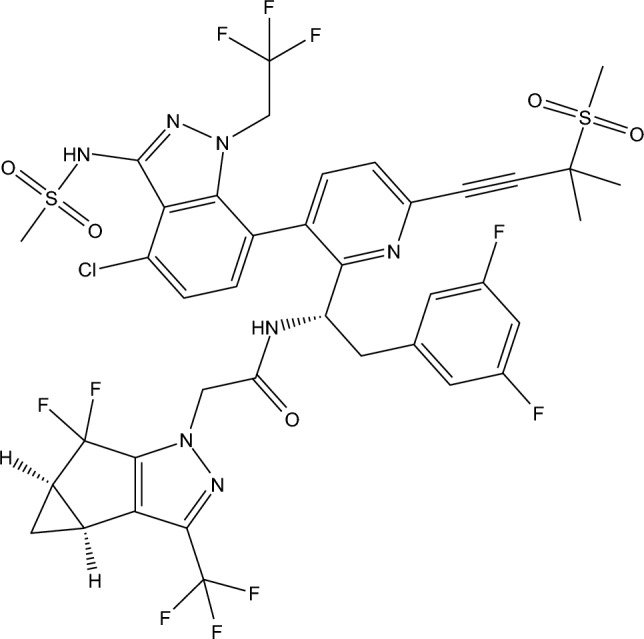
Chemical structure of lenacapavir

## Scientific Summary

### Pharmacodynamics

Lenacapavir selectively inhibits HIV-1 capsid function and therefore HIV-1 replication by binding directly to the interface between capsid protein subunits [4]. This inhibition occurs at multiple stages of the HIV replication lifecycle, including viral nuclear import (via inhibition of capsid-binding import co-factors), virion assembly (by compromising the stability of Gag and/or Gag-Pol polyproteins, which are crucial for virion assembly and within which capsid proteins are expressed), and capsid core assembly (by impairing its proper assembly) [7, 8].

In vitro data in HIV-1-infected cells showed lenacapavir to have half maximal effective concentrations (EC_50_) of 105 pmol/L in MT-4 cells, 32 pmol/L in primary human CD4^+^ cells, and 56 pmol/L in macrophages [8]. Lenacapavir also demonstrated antiviral activity against all major HIV-1 subtypes (including A, A1, AE, AG, B, BF, C, D, E, F, G and H) [8, 9]. HIV-1 variants with the capsid mutations L56I, M66I, Q67H, K70N, Q67H+N74S and Q67H+T107N each showed reduced susceptibility to lenacapavir in vitro (6 to > 3200-fold resistance vs wild type) [8].

In the ongoing, multinational phase II/III CAPELLA clinical trial, 8 of 19 patients receiving lenacapavir plus optimized background therapy (OBT) and assessed for resistance developed lenacapavir-associated capsid substitutions after 26 weeks of treatment, including M66I (6 patients; 1 with M66I+N74D), Q67H+K70H (1 patient), and K70H (1 patient) [10]. Relative to wild type, susceptibility to lenacapavir was reduced by 15-fold, 234-fold and 265-fold in patients with Q67H+K70R, M66I, and K70H mutations, respectively. All 8 cases of resistance to lenacapavir developed in patients on functional lenacapavir monotherapy, either due to poor adherence to OBT (*n* = 4) or no other fully active agents in the OBR (*n* = 4) [10]. In the ongoing phase II CALIBRATE trial assessing lenacapavir in treatment-naïve patients, 2 (of 157) patients had emergent resistant mutations occurring in the setting of likely or confirmed non-adherence to oral antiretroviral therapy, including Q67H+K70R and M184M/I in one patient (at week 10) and Q67H in the other (at week 54) [11].

The antiviral activity of lenacapavir was not affected in HIV-1 isolates with resistance mutations to protease inhibitors, nucleoside and nucleotide reverse transcriptase inhibitors (NRTIs), non-nucleoside reverse transcriptase inhibitors (NNRTIs), integrase strand transfer inhibitors (INSTIs), maturation inhibitors [12] and entry inhibitors [13]. Further in vitro data suggest that lenacapavir in combination with islatravir, rilpivirine or cabotegravir may have an additive antiviral effect, particularly at higher doses [14].

Lenacapavir had no clinically relevant effect on the QTcF interval, with no association seen between plasma concentrations of lenacapavir and changes in QTcF [4].

### Pharmacokinetics

The single-dose pharmacokinetics of oral lenacapavir are non-linear and less than dose-proportional over a dose range of 50–1800 mg, and those of SC lenacapavir are dose proportional over a dose range of 309–927 mg [4]. Peak plasma concentrations occur ≈ 4 h post-dose with oral lenacapavir and (due to slow release from the injection site) 84 days post-dose with SC lenacapavir; absolute bioavailability after administering oral lenacapavir is ≈ 6–10%. The pharmacokinetics of oral lenacapavir are unaffected by food [4].

In a population pharmacokinetic analysis, exposure to lenacapavir with respect to area under the concentration-time curve over a dosing interval (AUC_tau_), maximum plasma concentration (C_max_) and trough concentration (C_trough_) were 29–84% higher in heavily treatment-experienced patients infected with HIV-1 relative to individuals without HIV-1 [4].

Lenacapavir is not extensively metabolized; none of its circulating metabolites individually accounted for > 10% of total plasma exposure [4]. It is primarily metabolized via CYP3A4 and UGT1A1. After a single intravenous dose of radiolabelled lenacapavir in healthy individuals, 76% of the total radioactivity was found in faeces and < 1% from urine. The median half-life of lenacapavir was 10–12 days and 8–12 weeks with oral and SC administration, respectively [4].

Although lenacapavir exposure was found to be higher in patients with severe kidney impairment (estimated CrCl 15–29 mL/min) and moderate hepatic impairment compared with that in healthy individuals, these increases were not considered to be clinically meaningful [15, 16]. Dialysis is not expected to affect the exposure of lenacapavir as lenacapavir is ≈ 99.8% protein bound [4].

Because lenacapavir is a substrate of CYP3A, UGT1A1 and P-glycoprotein (P-gp), co-administration with strong inducers of these enzymes is contraindicated as they may reduce plasma lenacapavir concentrations, potentially impacting the therapeutic effect of lenacapavir and leading to the development of viral resistance [4]. Co-administration with strong inhibitors of all three enzymatic pathways (e.g. atazanavir/cobicistat) is not recommended as this may significantly increase plasma lenacapavir levels [4].

**Table:** Features and properties of lenacapavir

Alternative names	GS 6207; GS-6207-02; GS-714207; GS-HIV; GS-HIV Na; Lenacapavir sodium; Sunlenca
Class	Acetamides; alkynes; antiretrovirals; antivirals; chlorobenzenes; fluorinated hydrocarbons; pyrazoles; pyridines; sulfonamides; sulfones; HIV capsid inhibitor
Mechanism of action	Multistage, selective inhibitor of HIV-1 capsid function that binds directly to the interface between capsid protein subunits to inhibit HIV-1 replication
Route of administration	Oral, SC
Pharmacodynamics	EC_50_ 32–105 pmol/L in HIV-1-infected macrophages and MT-4 and CD4^+^ cells
	Antiviral activity demonstrated against all major HIV-1 subtypes (A, A1, AE, AG, B, BF, C, D, E, F, G and H)
	Antiviral activity unaffected in HIV-1 with resistance mutations to protease inhibitors, NRTIs, NNRTIs and INSTIs
	↓ susceptibility to lenacapavir seen in HIV-1 variants with L56I, M66I, Q67H, K70N, Q67H+N74S and Q67H+T107N mutations (6 to > 3200-fold resistance vs wild type)
Pharmacokinetics	T_max_ ≈ 4 h post-dose with oral lenacapavir, 84 days post-dose with SC lenacapavir
	Days 1–15 (oral lenacapavir 600 mg on days 1 and 2, 300 mg on day 8; SC lenacapavir 927 mg on day 15): C_max_ 69.6 ng/mL, AUC_tau_ 15,600 h∙ng/mL, C_trough_ 35.9 ng/mL
	Day 15 to end of month 6 (SC lenacapavir 927 mg): C_max_ 87 ng/mL, AUC_tau_ 250,000 h∙ng/mL, C_trough_ 32.7 ng/mL
	Steady state: C_max_ 97.2 ng/mL, AUC_tau_ 300,000 h∙ng/mL, C_trough_ 36.2 ng/mL
Adverse events	
Most frequent	Injection site reactions (swelling, pain, nodule, erythema, induration, pruritus, extravasation, discomfort, mass haematoma, oedema, and ulcer)
Common	Nausea
ATC codes	
WHO ATC code	J05-AX31 (lenacapavir)
EphMRA ATC code	J5C (HIV antivirals)
Chemical name	N-[(1R)-1-[3-[4-Chloro-3-(cyclopropylsulfonylamino)-1-(2,2-difluoroethyl)indazol-7-yl]-6-(3-methyl-3-methylsulfonylbut-1-ynyl)pyridin-2-yl]-2-(3,5-difluorophenyl)ethyl]-2-[(2R,4S)-9-(difluoromethyl)-5,5-difluoro-7,8-diazatricyclo[4.3.0.02,4]nona-1(6),8-dien-7-yl]acetamide

*EC*_*50*_ half maximal effective concentration, *INSTI* integrase strand transfer inhibitor, *NNRTI* non-nucleoside reverse transcriptase inhibitor, *NRTI* nucleotide reverse transcriptase inhibitor, *SC* subcutaneous

### Therapeutic Trials

Lenacapavir reduced HIV-1 viral load in patients with multidrug-resistant infection in the CAPELLA trial (NCT04150068; *n* = 72) [10]. After 15 days (i.e. at the end of the functional monotherapy period), 88% and 17% of lenacapavir and placebo recipients on failing antiretroviral therapy (i.e. cohort 1) had a decrease in plasma HIV-1 RNA of ≥ 0.5 log_10_ copies/mL from baseline [10].

Patients eligible for CAPELLA (age ≥ 12 years) had resistance to ≥ 2 retroviral medications from ≥ 3 of the 4 main drug classes (i.e. protease inhibitors, NRTIs, NNRTIs, INSTIs) and no more than 2 fully active antiretroviral drugs that could be combined from the 4 drug classes [10]. The first 36 enrolled patients with no response to previous antiviral therapy, as indicated by stable viremia (i.e. HIV-1 RNA levels reduced by < 0.5 log_10_ copies/mL) between screening and cohort allocation were placed in cohort 1 and randomized 2:1 to receive oral lenacapavir at the recommended dosage or placebo in addition to their failing antiviral therapy over a 15-day functional monotherapy period. During maintenance therapy (starting on day 15), lenacapavir recipients received SC lenacapavir at the recommended dosage plus OBT, while initial placebo recipients received oral lenacapavir at dosages of 600 mg/day on days 15 and 16 and 300 mg on day 22 before receiving SC lenacapavir plus OBT. All 36 patients in cohort 2 received lenacapavir plus OBT throughout the study and included those with HIV-1 RNA level reductions of ≥ 0.5 log_10_ copies/mL between screening and cohort allocation, as well as those eligible for (but enrolled after the filling of) cohort 1 [10].

After 26 days in cohorts 1 and 2, a viral load of < 50 copies/mL was seen in 81% and 83% of patients and < 200 copies/mL in 89% and 86% of patients, with mean changes in viral load of −2.58 ± 1.04 log_10_ copies/mL and −2.49 ± 1.34 log_10_ copies/mL from baseline [10]. Least-squares mean increases of 75 cells/mm^3^ and 104 cells/mm^3^ in CD4+ count were seen in the two cohorts [10]. More recent findings indicate that lenacapavir maintained its efficacy over long term treatment; after 52 weeks, 83% of patients in cohort 1 had a viral load of < 50 copies/mL (most patients in cohort 2 had not reached 52 weeks of treatment at the time of analysis) [17]. Lenacapavir therapy appeared to be similarly efficacious in patients considered more difficult to treat, including those with low CD4+ count, resistance to INSTI, no fully active agents, or no dolutegravir or darunavir in their OBT [18].

In the open-label phase II CALIBRATE study (*n* = 182), lenacapavir in combination with tenofovir alafenamide (TAF), bictegravir, or emtricitabine/TAF resulted in high viral suppression rates in treatment-naïve patients [11]. After 54 weeks, the majority of patients receiving lenacapavir combination therapy (85–90% across three treatment groups) and 92% of patients receiving bictegravir/emtricitabine/TAF had HIV-1 RNA levels of < 50 copies/mL [11]. Eligible patients, who had HIV-1 RNA counts of ≥ 200 copies/mL and CD4+ cell counts of ≥ 200 cells/µL, were randomized 2:2:2:1 to treatment groups 1–4; groups 1 and 2 received SC lenacapavir (at the recommended dosage) plus oral daily emtricitabine/TAF for 28 weeks, with group 1 receiving SC lenacapavir plus oral daily TAF as maintenance therapy beyond week 28 and group 2 receiving SC lenacapavir plus oral daily bictegravir as maintenance therapy beyond week 28, and group 3 received oral daily lenacapavir plus emtricitabine/TAF and group 4 received oral daily bictegravir/emtricitabine/TAF throughout the study [11]. Reductions in viral load were also seen with lenacapavir in combination with these antiretrovirals after 16 weeks and 28 weeks [11, 19].

**Table:** Key clinical trials of lenacapavir in HIV-1 infections

Drug(s)	Phase	Status	Location(s)	Identifier
Lenacapavir, emtricitabine/TAF, emtricitabine/TDF	III	Recruiting	South Africa, Uganda	NCT04994509, GS-US412-5624, DOH27-102021-6681, DOH27- 072021-6125, PURPOSE 1
Lenacapavir, emtricitabine/TDF, emtricitabine/TAF (USA only)	III	Recruiting	USA, Puerto Rico, South Africa	NCT04925752, GS-US528-9023, DOH27-102021-6681, PURPOSE 2
Lenacapavir, placebo	II/III	Active, no longer recruiting	Multinational	NCT04150068, EudraCT2019-003814-16, GS-US200-4625, JapicCTI205265, CAPELLA
Bictegravir/lenacapavir, stable baseline regimen (antiretrovirals)	II/III	Recruiting	Multinational	NCT05502341, EudraCT2022-500929-33-00, GS-US621-6289
Lenacapavir, emtricitabine/TAF, bictegravir, bictegravir/emtricitabine/TAF	II	Active, no longer recruiting	USA, Puerto Rico	NCT04143594, GS-US200-4334, CALIBRATE

*TAF* tenofovir alafenamide, *TDF* tenofovir disoproxil fumarate

### Adverse Events

In CAPELLA, oral and SC lenacapavir was generally well tolerated [10]. In the 15-week functional monotherapy period, 38% and 25% of (oral) lenacapavir and placebo recipients in cohort 1 experienced ≥ 1 adverse event (AE), none of which were serious or grade ≥ 3 in severity. Nausea was the only AE occurring in more than one lenacapavir recipient during this time (13% vs 0% with placebo). The most common AEs after 26 weeks of treatment with lenacapavir were injection site reactions, including pain (31%), swelling (31%), erythema (25%), nodule formation (24%) and induration (15%); 62% of patients experienced lenacapavir-related injection site reactions. Most injection site reactions were mild in severity and resolved within days. Other common AEs (incidence ≥ 10%) included nausea (12%), constipation (11%), diarrhoea (11%) and abdominal distention (10%), most of which were mild in severity and none of which were considered to be related to lenacapavir. Seven patients experienced serious AEs (SAEs), none of which were considered to be related to lenacapavir therapy. One patient with emergent lenacapavir resistance died at week 10. Laboratory abnormalities of grade ≥ 3 were reported in 28% of patients, including creatinine clearance (13%), creatinine (10%), glycosuria (6%), hyperglycaemia (fasting; 6%), aspartate aminotransferase (AST; 3%), direct bilirubin (3%) and proteinuria (3%) [10]. Lenacapavir continued to be generally well tolerated after 52 weeks of treatment in CAPELLA [17]. One patient discontinued lenacapavir after developing an injection site nodule (grade 1) 10 weeks after receiving a dose at 52 weeks [17].

Lenacapavir plus any of the assessed combination treatments in CALIBRATE was also generally well tolerated [11]. After 54 weeks in those who received SC lenacapavir, injection site reactions were mostly mild or moderate in severity and included erythema (27%), swelling (23%) and pain (19%). The most common non-injection site-related AEs were headache and nausea (13% each). No SAEs related to lenacapavir were reported [11].

### Ongoing Clinical Trials

The CAPELLA and CALIBRATE trials are ongoing. Two multinational phase III trials, PURPOSE 1 (NCT04994509) and PURPOSE 2 (NCT04925752), are currently recruiting; both will assess lenacapavir as a single agent for pre-exposure prophylaxis in individuals at risk of HIV-1 infection, with PURPOSE 1 focusing on adolescent girls and young women and PURPOSE 2 focusing on individuals assigned male at birth. Recruitment is also underway for a phase II/III trial, NCT05502341, which will assess the efficacy of oral bictegravir/lenacapavir in virologically suppressed, treatment-experienced individuals living with HIV on complex regimens switching from stable antiretroviral therapy versus continuing stable antiretroviral therapy.

## Current Status

Lenacapavir received its first approval on 22 August 2022 for use in combination with other antiretroviral(s) in adults with multi-drug resistant HIV infection, for whom it is otherwise not possible to construct a suppressive anti-viral regimen in the EU [5].

## Supplementary Information

Below is the link to the electronic supplementary material.

Supplementary file1 (PDF 575 KB)

## References

[1] Teeraananchai S, Chaivooth S, Kerr SJ (2017). Life expectancy after initiation of combination antiretroviral therapy in Thailand. Antivir Ther.

[2] Oh KS, Han E (2021). A comparison of medication adherence and viral suppression in antiretroviral treatment-naive patients with HIV/AIDS depending on the drug formulary. PLoS ONE.

[3] Shubber Z, Mills EJ, Nachega JB (2016). Patient-reported barriers to adherence to antiretroviral therapy: a systematic review and meta-analysis. PLoS Med.

[4] Gilead Sciences Inc. Lenacapavir (Sunlenca^®^): EU summary of product characteristics. 2022. https://ema.europa.eu/. Accessed 21 Sep 2022.

[5] Gilead Sciences Inc. Gilead announces first regulatory approval for Sunlenca^®^ (lenacapavir), the only twice-yearly HIV treatment option [media release]. 22 Aug 2022. https://www.gilead.com/.

[6] Gilead S. Gilead and Merck announce agreement to jointly develop and commercialize long-acting, investigational treatment combinations of lenacapavir and islatravir in HIV [media release]. 15 Mar 2021. http://www.gilead.com.

[7] Bester SM, Wei G, Zhao H (2020). Structural and mechanistic bases for a potent HIV-1 capsid inhibitor. Science.

[8] Link JO, Rhee MS, Tse WC (2020). Clinical targeting of HIV capsid protein with a long-acting small molecule. Nature.

[9] Callebaut C, VanderVeen L, Margot N. Activity and resistance characterization of the HIV capsid inhibitor lenacapavir [abstract no. 128]. In: 28th Conference on Retroviruses and Opportunistic Infections. 2021.

[10] Segal-Maurer S, DeJesus E, Stellbrink HJ (2022). Capsid inhibition with lenacapavir in multidrug-resistant HIV-1 infection. N Engl J Med.

[11] Gupta S, Sims J, Brinson C, et al. Lenacapavir as part of a combination regimen in treatment-naive people with HIV: week 54 results [abstract no. 138 plus oral presentation]. In: 29th Conference on Retroviruses and Opportunistic Infections. 2002.

[12] Margot N, Ram R, Rhee M (2021). Absence of lenacapavir (GS-6207) phenotypic resistance in HIV Gag cleavage site mutants and in isolates with resistance to existing drug classes. Antimicrob Agents Chemother.

[13] Margot N, Naik V, VanderVeen L. Absence of cross-resistance to lenacapavir in HIV entry inhibitor-resistant isolates [abstract no. 508]. In: 29th Conference on Retroviruses and Opportunistic Infections. 2022.

[14] Cilento ME, Ong YT, Tedbury PR (2022). Drug interactions in lenacapavir-based long-acting antiviral combinations. Viruses.

[15] Weber E, Graham H, West S, et al. Pharmacokinetics of lenacapavir in participants with severe renal impairment [abstract no. 434]. In: 29th Conference on Retroviruses and Opportunistic Infections. 2022.

[16] Jogiraju V, Begley R, Hindman J, et al. Pharmacokinetics of lenacapavir, an HIV-1 capsid inhibitor, in hepatic impairment [abstract no. 375]. In: 28th Conference on Retroviruses and Opportunistic Infections. 2021.

[17] Ogbuagu O, Segal-Maurer S, Brinson C (2022). Long-acting lenacapavir in people with multi-drug resistant HIV-1: week 52 results [abstract no. P006]. HIV Med.

[18] Stellbrink HJ, DeJesus E, Segal-Maurer S (2021). Subgroup efficacy analyses of long-acting subcutaneous lenacapavir in phase 2/3 in heavily treatment-experienced people with HIV (CAPELLA study). HIV Med.

[19] Gupta SK, Berhe M, Crofoot G (2021). Long-acting subcutaneous lenacapavir dosed every six months as part of a combination regimen in treatment-naive people with HIV: interim 16-week results of a randomized, open-label, phase 2 induction-maintenance study (CALIBRATE) [abstract no. OALB0302 plus presentation]. J Int AIDS Soc.

