# Supplementary material for: Lenacapavir: First Approval
Source: Drugs. 2022 Oct 22;82(14):1499–504. doi: 10.1007/s40265-022-01786-0 (PMC10267266; doi:10.1007/s40265-022-01786-0)
Supplement: Supplementary file 1 — Supplementary file1 (PDF 575 KB) [file 40265_2022_1786_MOESM1_ESM.pdf]

Drugs

PEER-REVIEWED FEATURE

Lenacapavir: Adis Evaluation

Key Points

- A long-acting HIV capsid inhibitor allowing bi-annual subcutaneous administration is being developed by Gilead Sciences Inc. for the treatment of HIV-1 infection
- Received its first approval on 22 August 2022 in the EU
- Approved in the EU for use in combination with other antiretroviral(s) in adults with multi-drug resistant HIV infection, for whom it is otherwise not possible to construct a suppressive anti-viral regimen

Summary

Lenacapavir (Sunlenca®) is a long-acting capsid inhibitor of human immunodeficiency virus type 1 (HIV-1) being developed by Gilead Sciences Inc.

It is available as an oral tablet and injectable solution, with the latter being a slow-release formulation to allow bi-annual subcutaneous administration.

In August 2022, lenacapavir received its first approval in the EU for use in combination with other antiretroviral(s) in adults with multi-drug resistant HIV infection, for whom it is otherwise not possible to construct a suppressive anti-viral regimen.

This summary represents the opinions of the author. For a full list of declarations, including funding and author disclosure statements, and copyright information, please see the full text online. © Springer Nature Switzerland AG 2022.
